# Incremental Value of Right Ventricular Outflow Tract Diameter in Risk Assessment of Chronic Heart Failure Patients with Implantable Cardioverter Defibrillators: Development of RVOTD-ICD Benefit Score in Real-World Setting

**DOI:** 10.31083/j.rcm2409269

**Published:** 2023-09-22

**Authors:** Hao Huang, Yu Deng, Sijing Cheng, Yu Yu, Xi Liu, Hongxia Niu, Xuhua Chen, Chi Cai, Min Gu, Wei Hua

**Affiliations:** ^1^Department of Cardiology, State Key Laboratory of Cardiovascular Disease, Fuwai Hospital, National Center for Cardiovascular Diseases, Chinese Academy of Medical Sciences and Peking Union Medical College, 100037 Beijing, China

**Keywords:** chronic heart failure, implantable cardioverter-defibrillator, life-threatening ventricular arrhythmia, right ventricular outflow tract diameter

## Abstract

**Background:**

Left ventricular ejection fraction (LVEF) remains the basic reference for the prevention of sudden cardiac death (SCD) patients, while right ventricular (RV) abnormalities have now been associated with SCD risk. A modified benefit assessment tool incorporating RV function parameters in consideration of implantable cardioverter defibrillators (ICD) insertion should be taken into account.

**Methods:**

We enrolled 954 chronic heart failure (CHF) patients (age 58.8 ± 13.1 years; 79.0% male) with quantitative measurements of right ventricular outflow tract diameter (RVOTD) before ICD implantation and then divided them according to the median level of RVOTD. The predictive value of RVOTD in life-threatening ventricular tachycardia (VT)/ventricular fibrillation (VF) vs. non-arrhythmic mortality (defined as death without prior sustained VT/VF), was evaluated respectively. Based on RVOTD and other identified risk factors, a simple risk assessment tool, RVOTD-ICD benefit score, was developed.

**Results:**

A higher RVOTD level was significantly associated with an increased risk of VT/VF (per 1 standard deviation (SD) increase, hazard ratio [HR], 1.22; 95% confidence interval [CI], 1.11–1.33; *p* = 0.002) but not non-arrhythmic mortality (per 1 SD increase, hazard ratio, 0.93; 95% CI, 0.66–1.33; *p* = 0.709) after multivariable adjustment. Three benefit groups were created based on RVOTD-ICD benefit score, which was calculated from VT/VF score (younger age, higher RVOTD, diuretic use, prior non-sustainable VT, prior sustainable VT/VF) and non-arrhythmic mortality scores (older age, renin-angiotensin-aldosterone system inhibitors use, diabetes, higher left ventricular end-diastolic diameter, New York Heart Association III/IV, higher N-terminal pro-B-type natriuretic peptide levels). In the highest RVOTD-ICD benefit group, the 3-year risk of VT/VF was nearly 8-fold higher than the corresponding risk of non-arrhythmic mortality (39.2% vs. 4.8%, *p* < 0.001). On the contrary, the 3-year risk of VT/VF was similar to the risk of non-arrhythmic mortality (21.9% vs. 21.3%, *p* = 0.405) in the lowest benefit group. RVOTD-ICD benefit score system yielded improvement in discrimination for VT/VF, non-arrhythmic mortality, and all-cause mortality than Multicenter Automatic Defibrillator Implantation Trial (MADIT)-ICD benefit score in this cohort.

**Conclusions:**

Higher RVOTD was associated with significantly increased risk of sustained VT/VF in CHF patients. A simple risk assessment tool incorporating RVOTD (RVOTD-ICD benefit score) could be generalized to ICD populations, and optimize the decision-making process of ICD implantation.

## 1. Introduction

Chronic heart failure (CHF) is a common end-stage heart disease and the leading cause of disability and death worldwide [1]. Despite improved management of cardiovascular diseases, the overall incidence of CHF is increasing in developed countries owing to the aging population [2]. Although patients with CHF usually die of various cardiac diseases, the specific causal mechanisms can be divided between sudden cardiac death (SCD) from arrhythmic events and non-SCD (NSCD) due to pump failure [3]. In the prevention of the former, implantable cardioverter-defibrillator (ICD) is a well-acknowledged treatment that can effectively monitor and terminate lethal ventricular arrhythmia [4].

Indications for ICD implantation are based mainly on a decreased left ventricular ejection fraction (LVEF; <35%) [4], while it may be insufficient as the sole criterion to stratify the risk of sudden arrhythmic death, especially since it represents only one-third of cases [5]. Furthermore, LVEF is associated with pump failure death, which cannot be directly prevented by ICD therapy. Thus, improved selection of patients at risk of SCD is required to bridge the gap between clinical evidence, avoidable device complications, and limited healthcare resources. In contrast, right ventricular (RV) dysfunction has shown reliable evidence for predicting adverse outcomes in different types of heart failure as well as SCD [6, 7, 8, 9, 10, 11]. The nature of this relationship between RV function parameters and ICD outcomes in patients with CHF warrants further investigation.

Moreover, previous studies focused on constructing risk assessment tools to facilitate risk stratification in primary prevention ICD recipients, while CHF patients with secondary implantation, many of whom have preserved (HFpEF) or mid-range (HFmrEF) LVEF, have received little attention. Risk stratification in these patients is essential to understanding the heterogeneity of disease development and prognosis. Therefore, the present study aimed to determine whether standard RV outflow tract diameter (RVOTD) measures could be easily obtainable predictors of ventricular arrhythmic events in CHF patients with varying functional statuses and be added to an individualized risk assessment tool in a real-world setting.

## 2. Materials and Methods

**Fig. 1.**
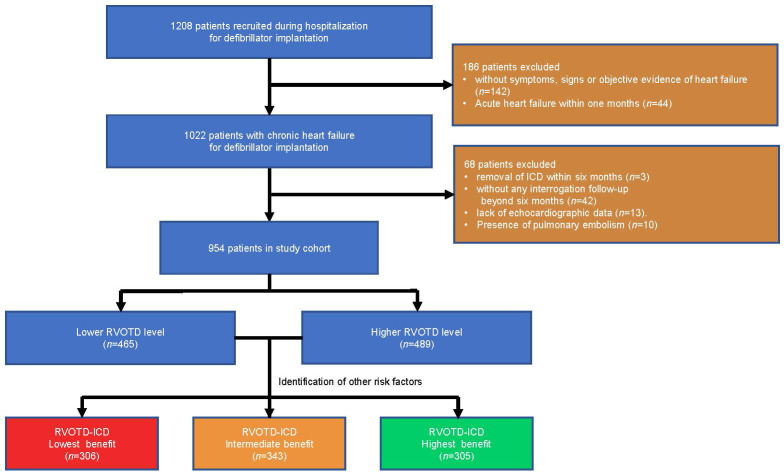
**Flow chart**. ICD, implantable cardioverter defibrillator; RVOTD, right ventricular outflow tract diameter.

We retrospectively enrolled consecutive patients with stable ambulatory CHF who underwent the implantation of a single- or dual-chamber ICD between January 1, 2010, and May 1, 2020. Patients were included if they presented with typical signs or symptoms of heart failure according to the latest European Society of Cardiology guidelines for the diagnosis of CHF [2]. For suspected heart failure patients with those symptoms/signs, natriuretic peptide measurement with N-terminal pro-B-type natriuretic peptide (NT-proBNP) ≥125 pg/mL or B-type natriuretic peptide (BNP) ≥35 pg/mL and abnormal findings hinted by electrocardiogram and echocardiography were used to confirm the diagnosis of CHF. In addition, medical history investigation, basic biochemical test and chest X-ray were comprehensively evaluated to differentiate CHF from other possible causes [2]. The exclusion criteria were: (1) without symptoms, signs or objective evidence of heart failure (n = 142); (2) acute heart failure within 1 month (n = 44); (3) ICD removal within 6 months (n = 3); (4) follow-up for interrogation less than 6 months (n = 42); (5) missing echocardiographic findings (n = 13); and (6) presence of pulmonary embolism (n = 10). Finally, 954 patients were included in this study (Fig. 1). The study complied with the Declaration of Helsinki and was approved by the Ethics Committee of Fuwai Hospital. All participants provided written informed consent.

### 2.1 Data Collection

Demographic characteristics, medication history and laboratory tests were collected from electronic medical records on admission. Two-dimensional echocardiographic examination was performed 3 days before ICD insertion by experienced sonographers and interpreted by well-trained cardiologists, using commercially available equipment. Sequential cardiac cycles were recorded during breath holding with stable electrocardiography tracing. Chamber dimensions and functional parameters were measured according to the American Society of Echocardiography and the European Association of Cardiovascular Imaging [12]. Right ventricular outflow tract diameter was measured from the anterior RV wall to the interventricular septal-aortic junction in a standard parasternal long-axis (PSLAX) end-diastole view, as depicted in **Supplementary Fig. 1** [13].

### 2.2 Follow-Up and Outcome Definitions

The follow-up period began on the first day after implantation. Device interrogation contained a review of the stored intracardiac electrograms 3 months later and every six to twelve months. The primary endpoint was the first appropriate shock triggered by ICD-monitored life-threatening ventricular tachycardia (VT)/ventricular fibrillation (VF) and undertreated SCD adjudicated by association-certified electrophysiologists. Shocks were determined appropriate if the preceding rhythm was classified as VT/VF. Inappropriate therapies and antitachycardia pacing (ATP) were excluded from the initial outcome. The secondary endpoint was non-arrhythmic mortality, defined as a composite of death or cardiac transplantation without exposure to any sustained VT/VF during follow-up. The survival status was obtained from medical health records or telephone calls until February 2022. The dates for the censoring of interrogation and death are not necessarily the same.

### 2.3 Model Development

Firstly, to explore an additional value of RVOTD based on existing VT/VF risk tools, the validated Seattle Proportional Risk Model (SPRM) was used as one of the basic models (male sex, younger age, no diabetes, lower left ventricular ejection fraction, lower systolic blood pressure, lower creatinine level, lower serum sodium level, better New York Heart Association functional class, higher body mass index and digoxin use) [14]. Another basic model is the Multicenter Automatic Defibrillator Implantation Trial (MADIT)-ICD benefit score, consisting of VT/VF score (LVEF ≤25%, atrial arrhythmia, heart rate >75 bpm, systolic blood pressure <140 mmHg, myocardial infarction, age <75 years, male, prior non-sustained VT) and non-arrhythmic mortality score (New York Heart Association ≥II, diabetes, body mass index <23 kg/$m^{2}$, atrial arrhythmia, LVEF ≤25%, and age ≥75 years) [15]. Cardiac resynchronization therapy-cardioverter defibrillator (CRT-D) recipients were not included considering that varying response rates of cardiac resynchronization therapy would confound their future effect on outcomes. Variable types and thresholds for categorization of numeric variables included in current analysis were basically the same as the original ones, and some corrections and explanations are stated as follows. Serum sodium values were analyzed as continuous units below 145 mEq/L, which is the upper limit of reference value in our hospital labs. When calculating MADIT-ICD scores, the points assigned for each variable were consistent with the original ones, too. All prior VT/VF events were analyzed when measuring MADIT-ICD VT/VF score.

Secondly, a new risk prediction model for ICD benefits incorporating RVOTD was introduced. We identified factors associated with increased risk for VT/VF, after accounting for non-arrhythmic mortality as a competing risk, and created a VT/VF risk score. Then, we used a similar method to construct the non-arrhythmic mortality risk score for death without a prior VT/VF as the endpoint. Next, we allocated each individual into a risk stratum by calculating a newly developed RVOTD-ICD benefit score that combined the VT/VF risk score and the non-arrhythmic mortality risk score for both outcomes. The whole population was separated into three benefit groups: (i) Highest benefit (highest VT/VF risk and lowest non-arrhythmic mortality risk), (ii) Intermediate benefit (higher VT/VF risk and lower non-arrhythmic mortality risk), and (iii) Lowest benefit (lowest risk of VT/VF and highest risk of non-arrhythmic mortality). Finally, we compared the RVOTD-ICD benefit score with MADIT-ICD benefit score by evaluating the overall survival benefit among these populations.

### 2.4 Statistical Analysis

Continuous data were presented as the median and the interquartile range (IQR) or mean and standard deviation. Categorical data were expressed as frequencies with percentages. Baseline characteristics of the lower and upper RVOTD patients were compared using χ^2^ test for categorical variables, the unpaired *t*-test for normally distributed continuous variables, and Kruskal‒Wallis test for continuous variables with nonnormal distribution.

Step 1—selection of RVOTD and other prognostic factors.

We performed analyses of the unadjusted cumulative incidence rates for VT/VF events and non-arrhythmic death, illustrated by Kaplan–Meier curves. Then, a multivariable Fine-Gray model, using non-arrhythmic mortality as a competitive risk and VT/VF as the endpoint, was used to adjust the effect of RVOTD in the final model. Variables in Seattle Proportional Risk Model, the MADIT-ICD VT/VF score and Non-arrhythmic Mortality Score, as well as other potential risk factors available for the entire cohort were candidate variables, whose *p* values less than 0.05 in univariable analysis were then included in the multivariable model to test for an independent association between outcomes and RVOTD. Backward selection was used based on Akaike Information Criterion (AIC) rule. The significance level for staying in the final model is 0.05. The same stepwise selective Cox regression for non-arrhythmic mortality was performed as for VT/VF. The follow-up time was calculated as the time between ICD implantation and the outcome events or censoring. Furthermore, RVOTD was added with all variables in SPRM and MADIT-ICD scores to assess the incremental value based on existing risk models. Interaction analysis were performed to examine potential heterogeneity of RVOTD between subgroups.

Step 2—development and validation of RVOTD-ICD benefit score.

Variables related to either VT/VF or non-arrhythmic death were selected based on step 1. To create a simple scoring method, numeric variables were categorized by the use of cut-off points. The age range was categorized by ten years. Log NT-proBNP was categorized by the quartile distribution. Thresholds for categorization of echocardiographic parameters were cut off by median. Each variable was then assigned a numeric value based on the relative value of its regression coefficient in the multivariate regression model. The prediction scores for VT/VF and non-arrhythmic mortality were separately validated by measuring discrimination using time-dependent receiver operating characteristic curve with 1000 randomly bootstrapped samples. Calibrations were assessed by comparing observed risk with the predictive risk of two RVOTD-ICD scores.

Given the VT/VF rate was approximately three times as non-arrhythmic death rate in the whole population, and in hope of creating positive scores, RVOTD-ICD benefit score was calculated as follows: 3 × VT/VF score – non-arrhythmic death score + 50, in which higher score denotes a higher long-term benefit from ICD implantation. The cohort was trichotomized into three groups based on patient-specific risk for VT/VF and non-arrhythmic mortality measured as ICD benefit score. Within each group, we used cumulative incidence function curves to illustrate both outcomes.

Step 3—comparison with MADIT-ICD benefit score.

ROC curve analysis using nonparametric estimates of the area under the curve (AUC) was performed to compare the predictability of VT/VF score and non-arrhythmic death score between MADIT-ICD and RVOTD-ICD models. Patient discrimination and reclassification were also evaluated using continuous net reclassification improvement (cNRI). Finally, the clinical usefulness and net benefit of both benefit score systems were estimated with decision curve analysis to test whether the new model would stratify long-term risk of whole ICD population in different ranges of threshold probabilities.

SPSS version 26.0 (IBM Corp., Armonk, NY, USA) and R version 4.1.2 (R Foundation for Statistical Computing, Vienna, Austria) were used for statistical analyses. Missing data were handled by multiple imputations. A two-sided *p*-value < 0.05 was considered statistically significant unless specified otherwise.

## 3. Results

### 3.1 Study Population

The 954 total ICD recipients were divided according to median RVOTD (23 mm) (Fig. 1). The population was on average 58.8 years old, and predominantly male (79.0%). The ICD manufacturers included Medtronic, Abbott, Biotronik, and Boston Scientific. A total of 604 patients underwent implantation of a single-chamber ICD. **Supplementary Fig. 2 **presents the distribution of RVOTD in the cohort. The baseline characteristics of the patients are shown in Table 1. Patients with a higher median RVOTD had a higher body mass index. They were more likely to suffer from atrial fibrillation and less likely to suffer from coronary arterial disease. In terms of echocardiographic parameters, they had higher left atrial diameters, but the left ventricular end-diastolic diameter and LVEF were similar. Additionally, they were more likely to be prescribed digoxin, and their erythrocyte sedimentation rates and lactic dehydrogenase concentrations were higher.

**Table 1.** **Baseline characteristics in patients with lower or higher levels of right ventricular diameters**.

		All (n = 954)	RVOTD <23 mm (n = 465)	RVOTD ≥23 mm (n = 489)	*p* value
Age		58.79 (13.08)	59.46 (13.15)	58.15 (13.00)	0.121
Female		200 (21.0)	123 (26.5)	77 (15.7)	<0.001
Body mass index (kg/$m^{2}$)		24.85 (3.56)	24.47 (3.39)	25.21 (3.69)	0.001
Heart rate (bpm)		68.74 (13.91)	68.21 (12.91)	69.25 (14.80)	0.247
NYHA class					0.913
	I/II	582 (61.0)	285 (61.3)	297 (60.7)	
	III/IV	372 (39.0)	180 (38.7)	192 (39.3)	
Smoking		439 (46.0)	217 (46.7)	222 (45.4)	0.743
Alcohol use		347 (36.4)	158 (34.0)	189 (38.7)	0.152
Prior VT		796 (83.4)	386 (83.0)	410 (83.8)	0.796
Prior Sustain VT/VF		636 (66.7)	306 (65.8)	330 (67.5)	0.679
Syncope		416 (43.6)	193 (41.5)	223 (45.6)	0.226
Frequent PVCs		419 (43.9)	208 (44.7)	211 (43.1)	0.669
Diabetes mellitus		190 (19.9)	85 (18.3)	105 (21.5)	0.249
Coronary arterial disease		451 (47.3)	242 (52.0)	209 (42.7)	0.005
Atrial fibrillation		285 (29.9)	115 (24.7)	170 (34.8)	0.001
Atrioventricular block		120 (12.6)	52 (11.2)	68 (13.9)	0.242
Stroke		62 (6.5)	29 (6.2)	33 (6.7)	0.85
Hyperlipidemia		473 (49.6)	244 (52.5)	229 (46.8)	0.093
eGFR <60 mL/min/1.73 $m^{2}$		225 (23.6)	109 (23.4)	116 (23.7)	0.979
Hyperuricemia		96 (10.1)	40 (8.6)	56 (11.5)	0.175
Left ventricular mass index		150.21 (52.13)	153.04 (53.88)	147.51 (50.33)	0.101
LVEDD		60.69 (10.78)	60.22 (10.72)	61.13 (10.83)	0.192
LAD (mean (SD))		43.51 (7.92)	42.04 (6.52)	44.90 (8.84)	<0.001
LVEF (mean (SD))		41.76 (13.80)	41.73 (13.77)	41.80 (13.84)	0.94
RAAS inhibitors		676 (70.9)	332 (71.4)	344 (70.3)	0.775
β-blocker		865 (90.7)	431 (92.7)	434 (88.8)	0.048
Calcium channel blockers		95 (10.0)	51 (11.0)	44 (9.0)	0.364
Diuretic		691 (72.4)	333 (71.6)	358 (73.2)	0.632
Mineralcorticoid receptor antagonist		627 (65.7)	308 (66.2)	319 (65.2)	0.797
Digoxin		231 (24.2)	99 (21.3)	132 (27.0)	0.048
Antiarrhythmic drugs		574 (60.2)	285 (61.3)	289 (59.1)	0.532
NT-proBNP		1570.83 (2171.68)	1362.14 (1926.87)	1769.27 (2366.20)	0.054
LDH		205.43 (76.14)	202.69 (81.94)	208.02 (70.15)	0.020
ESR		11.10 (12.37)	12.17 (13.46)	10.09 (11.15)	0.006
hs-TnI		0.22 (1.01)	0.25 (1.25)	0.19 (0.70)	0.913
hs-CRP		3.52 (3.98)	3.41 (3.98)	3.62 (3.98)	0.132

Values are the mean (SD) or n (%). eGFR, estimated glomerular filtration rate; ESR, erythrocyte sedimentation rate; hs-CRP, high-sensitivity C-reactive protein; hs-TnI, high-sensitivity troponin I; LAD, left atrial diameter; LDH, lactic dehydrogenase; LVEDD, left ventricular end-diastolic diameter; LVEF, left ventricular ejection fraction; NSVT, non-sustained ventricular tachycardia; NT-proBNP, N-terminal pro-B-type natriuretic peptide; NYHA, New York Heart Association; PVCs, premature ventricular complexes; RAAS, renin-angiotensin-aldosterone system; VF, ventricular fibrillation; VT, ventricular tachycardia; RVOTD, right ventricular outflow tract diameter.

### 3.2 Association between RVOTD and Outcomes

**Fig. 2.**
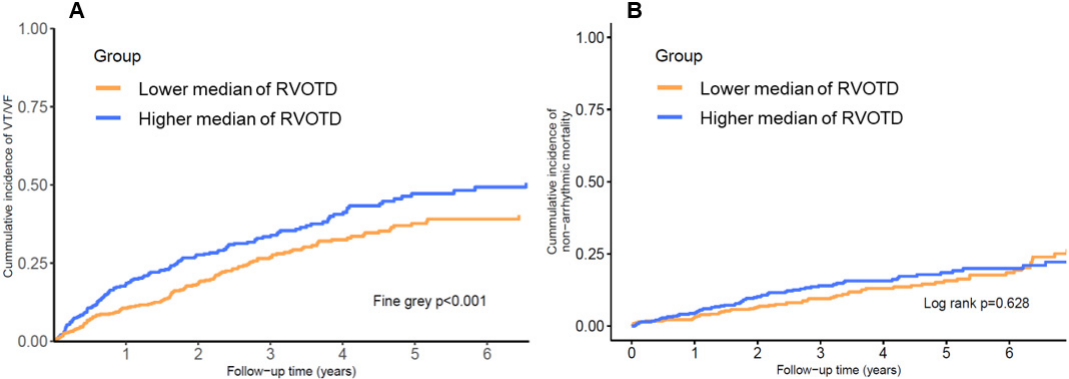
**Incidence of VT/VF (A) and non-arrhythmic mortality (B) in patients with lower or higher right ventricular outflow tract diameters**. RVOTD, right ventricular outflow tract diameter; VT, ventricular tachycardia; VF, ventricular fibrillation.

During a median interrogation follow-up of 2.83 years (interquartile range: 1.33–5.27 years) and median death follow-up of 3.85 years (interquartile range: 2.14–6.37 years), 285 patients experienced appropriate ICD shock (29.9%) or SCD (0.2%), and 140 patients died without experiencing any sustained VT/VF since implantation (14.7%). In the unadjusted time-to-event curves, patients with a higher median RVOTD had a higher cumulative incidence of VT/VF events than their lower counterparts (Fig. 2A, Table 2) (hazard ratio [HR], 1.48; 95% confidence interval [CI], 1.17–1.87; *p* = 0.001). However, the non-arrhythmic mortality risk was not significantly different between the two groups (Fig. 2B, Table 2; HR, 1.09; 95% CI, 0.78–1.52; *p* = 0.628). A multivariable model fully adjusted for all possible confounders in the univariate analysis (**Supplementary Table 1**) showed consistent adverse effects of RVOTD in VT/VF events (HR per standard deviation (SD): 1.22; 95% CI, 1.11–1.33; *p* = 0.002). Receiver operating characteristic curve analysis confirmed the predictive value of RVOTD for VT/VF events (area under the curve [AUC], 0.61; 95% CI, 0.55–0.66; *p* < 0.001), with the best cutoff value at 27 mm. The fully adjusted Seattle Proportional Risk Model and Multicenter Automatic Defibrillator Implantation Trial–Implantable Cardioverter-Defibrillator (MADIT-ICD) models also suggested that RVOTD was a robust indicator of VT/VT events, both as categorical and continuous variables (Table 2).

**Table 2.** **Association between right ventricular outflow tract diameter levels and outcomes**.

Group		Event rate (per 100 person-year)	Unadjusted HR (95% CI)	*p *value	SPRM adjusted* HR (95% CI)	*p* value	MADIT-ICD ${\mathrm{adjusted}}^{†}$ HR (95% CI)	*p* value
VT/VF events		12.20 (10.82–13.70)						
	Lower median	9.61 (7.99–11.46)	Reference		Reference		Reference	
	Higher median	15.39 (13.11–17.96)	1.48 (1.17–1.87)	0.001	1.45 (1.15–1.84)	0.002	1.45 (1.04–1.69)	0.020
	Per 1 SD increase		1.23 (1.12–1.35)	<0.001	1.20 (1.09–1.32)	<0.001	1.19 (1.08–1.31)	<0.001
Non-arrhythmic mortality		4.07 (3.43–4.81)						
	Lower median	3.96 (3.08–4.99)	Reference		Reference		Reference	
	Higher median	4.20 (3.28–5.31)	1.09 (0.78–1.52)	0.628	1.15 (0.82–1.62)	0.424	1.06 (0.76–1.49)	0.715
	Per 1 SD increase		0.97 (0.82–1.14)	0.680	1.08 (0.90–1.30)	0.383	1.00 (0.85–1.19)	0.969

*SPRM adjusted model initially included RVOTD and all the variables in Seattle Proportional Risk Model (male sex, younger age, no diabetes, lower left ventricular ejection fraction, systolic blood pressure, lower creatinine level, serum sodium level, better NYHA functional class, body mass index and digoxin use). Final variables were backward selected based on AIC rule for each outcome. ^†^For VT/VF events, MADIT-ICD adjusted model initially included RVOTD and all the variables in MADIT-ICD VT/VF score (LVEF <25%, atrial arrhythmia, heart rate >75 bpm, SBP <140 mmHg, myocardial infarction, age <75 years, male, and prior sustained VT/VF), which were then backward selected based on AIC rule; For non-arrhythmic mortality, MADIT-ICD adjusted model initially included RVOTD and all the variables in MADIT-ICD non-arrhythmic mortality score (NYHA ≥II, diabetes, BMI <23 kg/$m^{2}$, atrial arrhythmia, LVEF ≤25%, age ≥75), which were then backward selected based on AIC rule. LVEF, left ventricular ejection fraction; NYHA, New York Heart Association; VF, ventricular fibrillation; VT, ventricular tachycardia; RVOTD, right ventricular outflow tract diameter; SPRM, Seattle Proportional Risk Model; BMI, body mass index; MADIT, Multicenter Automatic Defibrillator Implantation Trial; ICD, implantable cardioverter defibrillator; SD, standard deviation; AIC, Akaike Information Criterion; HR, hazard ratio; SBP, systolic blood pressure.

Subgroup analyses demonstrated that the link between RVOTD and VT/VF was homogenous across various subgroups of patients, particularly among patients with or without left ventricle hypertrophy or different LVEF groups (**Supplementary Fig. 3**). Interestingly, the risk of RVOTD may be even higher in those who were not treated with guideline-directed pharmacotherapy, including renin-angiotensin-aldosterone system (RAAS) inhibitors, β-blockers, and mineralocorticoid receptor antagonists (*p* for interaction <0.05). A sensitivity analysis, including monitoring ATP as the primary endpoint, revealed a robust association between RVOTD and ventricular tachyarrhythmia (**Supplementary Table 2**).

### 3.3 Development and Validation of RVOTD-ICD Benefit Score

Together with a higher RVOTD, younger age, diuretic use, prior non-sustained VT (NSVT), and prior sustained VT/VF were eventually identified as having an increased risk of VT/VF in the Fine-Gray regression model (Table 3). The AUC of the RVOTD-ICD VT/VF score of 1, 2, and 3 years were 0.64 (95% CI, 0.59–0.68), 0.64 (95% CI, 0.59–0.68), and 0.62 (95% CI, 0.57–0.66), respectively (**Supplementary Table 3**). On the other hand, six factors were identified as predictors of non-arrhythmic mortality: older age, diabetes, higher left ventricular end-diastolic diameter (LVEDD), New York Heart Association (NYHA) class III/IV and higher NT-proBNP quartile were associated with increased risk of non-arrhythmic mortality, whereas RAAS inhibitors use was related to reduced risk (Table 3). The AUC of the RVOTD-ICD non-mortality score of 1, 2, and 3 years were 0.81 (95% CI, 0.73–0.88), 0.75 (95% CI, 0.69–0.81), and 0.78 (95% CI, 0.73–0.83), respectively (**Supplementary Table 3**). Calibration curves showed that both the RVOTD-VT/VF score and non-mortality score fit well with the corresponding risks at 1, 2, and 3 years (**Supplementary Fig. 4**). A consistent difference in the VT/VF rates was also observed between the primary and secondary prevention populations (**Supplementary Table 4**).

**Table 3.** **Regression models cooperated with right ventricular outflow tract diameter and other variables and their corresponding point for newly developed RVOTD-ICD VT/VF and non-arrhythmic mortality risk score**.

Variable		VT/VF score				Non-arrhythmic mortality score			
		Coefficient	HR	*p* value	Score	Coefficient	HR	*p* value	Score
Age per 10 years		–0.20	0.82	<0.001		0.22	1.24	0.004	
	<45				+2				–2
	45 ≤ age < 55				+1				–1
	55 ≤ age < 65	Reference	Reference		0	Reference	Reference		0
	65 ≤ age < 75				–1				+1
	≥75				–2				+2
RVOTD ≥23		0.37	1.45	0.002	+2				
Diuretics		0.40	1.49	0.005	+2				
Prior NSVT		0.49	1.63	0.041	+2				
Prior sustain VT/VF		0.67	1.96	<0.001	+3				
RAAS inhibitors						–0.46	0.63	0.010	–2
Diabetes						0.47	1.60	0.019	+2
LVEDD ≥68						0.57	1.78	0.003	+3
NYHA III/IV						0.66	1.93	<0.001	+3
NT-proBNP in quartile									
	<392					Reference	Reference		0
	392 ≤ NT-proBNP < 910					1.22	3.39	0.014	+6
	910 ≤ NT-proBNP < 1887					1.80	6.03	<0.001	+8
	≥1887					2.14	8.46	<0.001	+10

LVEDD, left ventricular end-diastolic diameter; NSVT, non-sustained ventricular tachycardia; NT-proBNP, N-terminal pro-B-type natriuretic peptide; NYHA, New York Heart Association; RAAS, renin-angiotensin-aldosterone system; VF, ventricular fibrillation; VT, ventricular tachycardia; RVOTD, right ventricular outflow tract diameter; HR, hazard ratio; ICD, implantable cardioverter defibrillator.

**Fig. 3.**
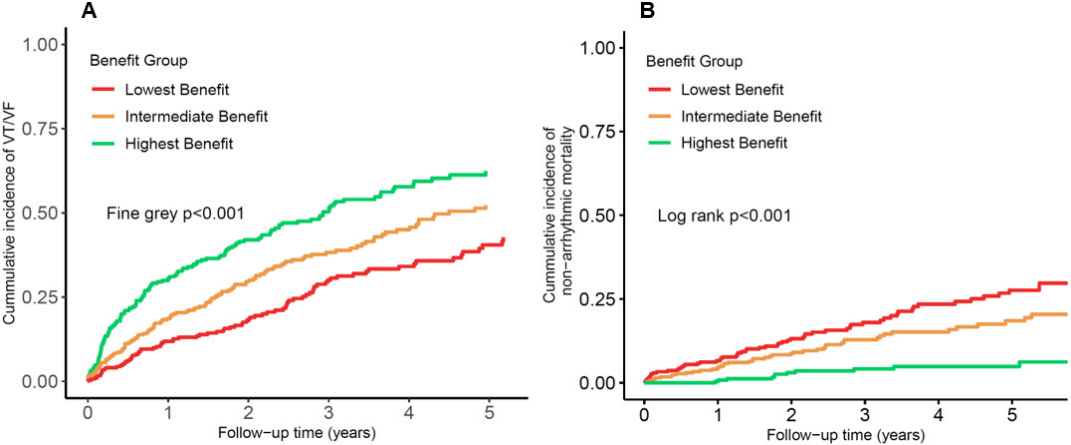
**Incidence of VT/VF (A) and non-arrhythmic mortality (B) among the three RVOTD-ICD benefit score groups**. ICD, implantable cardioverter defibrillator; RVOTD, right ventricular outflow tract diameter; VT, ventricular tachycardia; VF, ventricular fibrillation.

The RVOTD-ICD benefit score was then calculated and displayed a normal distribution (Shapiro–Wilk test, *p* = 0.109; **Supplementary Fig. 5**). According to the benefit score, this population was divided into the high benefit group (>61), intermediate benefit group (54–61), and lowest benefit group (<54). Fig. 3A,B illustrates the cumulative incidence curves for the observed risk of VT/VF and non-arrhythmic mortality in each of the three RVOTD-ICD benefit groups. The benefit score divided groups well stratified the whole population in both the risk of VT/VF (Fine-Gray, *p* < 0.001) and non-arrhythmic mortality (log rank, *p* < 0.001). The ATP-included endpoints displayed similar results (**Supplementary Fig. 6**). The predicted risks in the RVOTD-ICD benefit groups are shown in Table 4. In the highest RVOTD-ICD benefit group, the 3-year VT/VF risk was approximately 8-fold higher than the corresponding risk of non-arrhythmic mortality (39.2% vs. 4.8%, *p* < 0.001, respectively). In the intermediate group, the 3-year VT/VF risk was also higher than the risk of non-arrhythmic mortality; however, the difference diminished (29.4% vs. 10.9%, *p* < 0.001). In contrast, the 3-year risk of VT/VF was similar to the risk of non-arrhythmic mortality (21.9% vs. 21.3%, *p* = 0.405) in the lowest RVOTD-ICD benefit group.

**Table 4.** **Predicted VT/VF and non-arrhythmic mortality risk by RVOTD-ICD benefit groups**.

RVOTD-benefit group		At 1 year			At 2 years			At 3 years		
		VT/VF	Non-arrhythmic mortality	*p* value	VT/VF	Non-arrhythmic mortality	*p* value	VT/VF	Non-arrhythmic mortality	*p* value
Highest benefit group										
	Predicted mean rate (%)	19.5	1.4	<0.001	30.4	3.2	<0.001	39.2	4.8	<0.001
	Predicted range (%)	19.0–20.1	1.2–1.6		29.6–31.2	2.9–3.6		38.2–40.1	4.2–5.4	
Intermediate benefit group										
	Predicted mean rate (%)	14.1	3.3	<0.001	22.4	7.5	<0.001	29.4	10.9	<0.001
	Predicted range (%)	13.7–14.5	3.0–3.6		21.9–23.0	6.9–8.2		28.7–30.2	10.0–11.9	
Lowest benefit group										
	Predicted mean rate (%)	10.2	6.7	<0.001	16.4	15.0	0.007	21.9	21.3	0.405
	Predicted range (%)	9.9–10.5	6.2–7.3		16.0–16.9	13.9–16.2		21.2–22.5	19.8–22.8	

ICD, implantable cardioverter defibrillator; RVOTD, right ventricular outflow tract diameter; VT, ventricular tachycardia; VF, ventricular fibrillation.

### 3.4 Comparison with MADIT-ICD Benefit Score

**Fig. 4.**
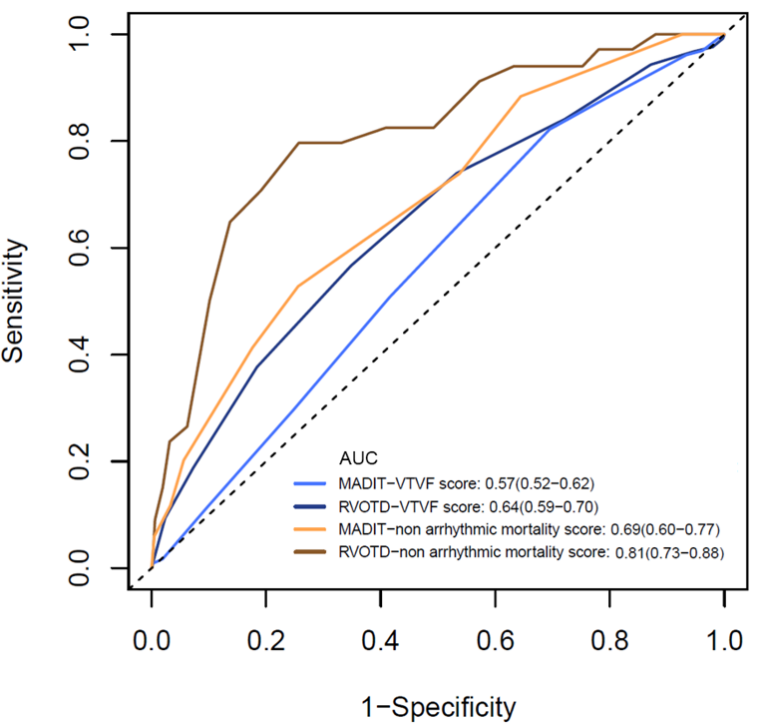
**Receiver Operating Characteristic Curve for the MADIT- and RVOTD-VT/VF score and non-arrhythmic mortality score**. RVOTD, right ventricular outflow tract diameter; VT, ventricular tachycardia; VF, ventricular fibrillation; MADIT, Multicenter Automatic Defibrillator Implantation Trial.

MADIT-ICD VT/VF, non-arrhythmic, and benefit scores were calculated as in the original research. The time-dependent receiver operating characteristic curve (Fig. 4) manifested that RVOTD-ICD VT/VF score yielded a more accurate prediction of both 1-year VT/VF (0.64 vs. 0.57, *p* = 0.029) and non-arrhythmic mortality (0.81 vs. 0.69, *p* = 0.006). NRI analysis also revealed that the RVOTD-ICD VT/VF score and non-arrhythmic mortality score could better reclassify 16.1% and 42.4% of patients for 1-year outcomes, respectively. The improvement was consistent in the two- and three-year observations shown in **Supplementary Table 3**.

Finally, decision curve analysis was applied to facilitate the comparison of long-term survival benefits between the scores (**Supplementary Fig. 7**). In the 5-year analysis, the RVOTD-ICD benefit score provided a larger net benefit across the range of all-cause mortality risk than the MADIT-ICD score. For example, at a threshold of 30% death risk, the RVOTD-ICD benefit score identified 4.4% additional all-cause mortality compared with the MADIT-ICD benefit score, without increasing the number of false positives (**Supplementary Table 5**).

## 4. Discussion

As far as we know, this is the first study to confirm the association between increased right ventricular outflow tract diameter and the risk of life-threatening ventricular arrhythmia in an ICD population with different causes and statuses of CHF. In addition, we provide a revised score system that could help identify CHF patients who more tend to benefit from ICD therapy in both primary and secondary prevention populations by evaluating individualized risk for life-threatening VT/VF and the competing risk of non-arrhythmic mortality. The results showed that RVOTD was significantly associated with VT/VF in multivariable analysis, independent of LV dysfunction, and RVOTD-ICD benefit score could better stratify arrhythmic-specific risk compared with MADIT-ICD score.

### 4.1 Current Risk Stratification of SCD

The fact that only 7–30% of ICD recipients for primary prevention in clinical trials received appropriate shocks suggests the need for improved SCD risk stratification [16]. Among the many clinical variables proposed as potential predictors of SCD [17], LVEF is a nearly exclusive marker used in the clinical decision of ICD implantation because of its simplicity and reliability, which has been substantiated in several randomized control trials [18, 19, 20]. However, in many observational studies, most patients with SCD had normal or mildly reduced LVEF [5]. The sole use of LVEF may be underqualified for the identification of patients at risk of SCD in different cardiac conditions, especially in non-ischemic cardiomyopathy [20, 21].

Moreover, previous studies have focused on constructing risk assessment tools to facilitate risk stratification in primary prevention ICD recipients, while CHF patients with secondary implantation were rarely taken seriously, probably based on the myth that these patients would certainly benefit from ICD. However, in consideration of cost-effectiveness, especially in underdeveloped areas, those with secondary indications are the majority of ICD recipients in real-world settings. Still, prior studies have failed to identify a specific group that exhibits a higher or lower risk of recurrence of potentially life-threatening ventricular arrhythmias among this population [22, 23]. More importantly, another thought-provoking basis for studying patients with CHF both with and without prior sustained VT/VF is that other causes of mortality that are not arrhythmic-related or even noncardiac play an increasing role in HFpEF [24], which is unavoidably underrepresented in the primary prevention population. In addition, efficacious medications for heart failure with reduced ejection fraction have been less so at higher LVEF ranges, only decreasing the risk of HF hospitalization but not cardiovascular death in HFpEF. This reflects the burden of noncardiac comorbidities as LVEF increases and emphasizes the complicated cardiac and noncardiac mechanisms underpinning HFpEF. Therefore, weighing between VT/VF and non-arrhythmic mortality needs to be individually conducted to appropriately evaluate the potential benefit of ICD in patients with CHF across LVEF. Our study concluded that LVEF and left atrial diameter were more competitive in predicting non-arrhythmic mortality than life-threatening ventricular arrhythmia in patients with CHF. Consequently, this phenomenon calls for other specific arrhythmogenic predictors for precise ICD implantation.

### 4.2 RV Dysfunction and Ventricular Arrhythmia

Recent studies have attached importance to RV function as a determinant of arrhythmic outcomes, including SCD and ventricular tachyarrhythmia [11, 25, 26, 27, 28, 29]. RV dysfunction (usually assessed by RV ejection fraction) is independently predictive of sudden cardiac arrest or appropriate ICD therapy, even among those with an LVEF >35% [11, 26, 27], which indicates that RV dysfunction has the potential to enhance the current approach to SCD risk stratification beyond left heart function. The possible mechanism by which RV involvement leads to the occurrence of VA/SCD has not been elucidated. Macro reentry, mainly due to a conduction delay within the scar zone, is frequently noted in ischemic cardiomyopathy. RV stretch and volumetric load would prolong cardiac repolarization and refractoriness, which may create an unstable electrophysiological substrate, giving rise to an enhanced propensity for stretch-triggered or stretch-mediated ventricular arrhythmias [30].

Surprisingly, this study showed that RVOTD was superior to LVEDD for arrhythmia risk stratification. The response of RV to disease is a consequence of various combinations of volume overload and/or pressure, as well as intrinsic myocardial deficits, where the predominant abnormality may determine the clinical presentation and course. Because the vast majority of patients had left-sided heart failure and acute pulmonary arterial hypertension was excluded in this study, additional RV involvement may be regarded as a sensitive barometer of any “downstream pathology” affecting RV afterload due to abnormal pulmonary vasculature, or most likely, increased LV filling pressures [31], which appears to identify a group with a high risk of ventricular tachyarrhythmia.

Although RV systolic function is an important predictor of cardiovascular outcomes, the complex morphology of the RV and the mechanics of motion make it difficult to obtain accurate and reproducible measurements [32]. Since the assessment of RV function is not currently routine before ICD implantation, many echocardiographic measures related to RV function were not available for every patient in this study. Nonetheless, we proved that an integrated record of right ventricular diameter was useful enough to reflect RV stretch and loaded volume to a huge extent and undoubtedly had a significant implication on the prognosis of malignant arrhythmic events.

It should be noted that the long-term effect of the proarrhythmic action of RVOTD was consistent in most clinical subgroups, including a history of cardiovascular disease across LVEF strata, indicating that RVOTD could serve as an incremental predictor of SCD in patients with reduced systolic function, whose risk of SCD has already increased, as well as in patients with preserved LVEF. The exception lies in the use of three foundational drugs for the treatment of CHF: RAAS inhibitors, β-blockers, and mineralocorticoid receptor antagonists. Although patients with a higher RVOTD experienced more VT/VF events regardless of whether they were taking anti-HF medications, the former were clearly less influenced by a larger RV at the time of implantation. It could be speculated that these drugs improve cardiac remodeling in both ventricles, and thus, improve arrhythmic outcomes. Additionally, for those who cannot tolerate anti-HF medications, cardiac pump function is usually not able to maintain blood pressure. Consequently, capacity-sensitive RV tend to expand, which further increases the risk of long-term arrhythmic events.

Several baseline characteristics, such as atrial fibrillation and digoxin use, were imbalanced between the higher and lower RVOTD groups, which may have confounded the proarrhythmogenic effect of RVOTD. However, univariable and multivariable adjustments of these characteristics minimized the study bias. Moreover, during every interrogation, the preceding rhythm of each previous shock was examined through an intracavitary electrogram, thus preventing the mistake that inadequate shocks for atrial tachyarrhythmia were regarded as endpoints.

### 4.3 RVOTD-ICD Benefit Score

In light of the MADIT-ICD benefit score, the new RVOTD-ICD benefit score could be more easily calculated manually and used for decision-making regarding the need for SCD prevention. In this population, the benefit of ICD was obvious in the first two years among all candidates, while the efficacy started dividing in the third year. The highest RVOTD-ICD benefit group comprised patients with the highest predicted risk for VT/VF and the lowest predicted risk for non-arrhythmic mortality; hence, the absolute need to receive an ICD. In the intermediate benefit group, the 3-year risk of VT/VF was still nearly three times higher than the corresponding risk of non-arrhythmic mortality. Thus, they should also be considered for ICD prevention, combined with concomitant treatment of associated comorbidities, to reduce the risk of non-arrhythmic death. Although the risk of experiencing three-year VT/VF is still over 20% to warrant ICD implantation in the lowest benefit group, the significant difference from the risk of non-arrhythmic mortality vanishes, suggesting that a personalized approach to device implantation should be considered with more focus on the management of comorbidities to maximize the benefit from ICD. Furthermore, this score can be extrapolated to patients with previous ventricular tachycardia or fibrillation, who account for a large proportion of patients in real-world situations. Even though ICD implantation seems inevitable for these patients, the RVOTD-ICD benefit score could still serve as a reference for individualized follow-up, including the frequency and emphasis of interrogation, and for more urgent ICD treatment, prior to the progression of more advanced risk factors associated with non-arrhythmic mortality.

Apart from the RVOTD identified in this study, one of the most recognized risk factors associated with ICD efficacy is age, as younger patients have a higher probability of appropriate ICD therapy [14, 15, 23, 33]. Prior NSVT is another established hazard for VT/VF risk [34]. This component and prior sustained VT/VF were assigned different points in the RVOTD-ICD VT/VF score. A large prospective study supported the extension of ICD to a selected population with prior NSVT from underrepresented geographies [35]. Diuretics have not been shown to reduce all-cause mortality in patients with CHF. Instead, we indicated that the need for diuretics in patients with CHF implies worse prognosis. It is also worth noting that thiazide and loop diuretics may cause electrolyte abnormalities and concomitant drug-induced arrhythmias.

With regard to non-arrhythmic mortality, diabetes [36] and a higher NYHA class [37] are widely acknowledged to present a high risk for non-arrhythmic mortality associated with various comorbidities. A larger left ventricular dimension, featuring left dysfunction, showed a better determination of pump failure death owing to its direct pump effect [38]. RAAS inhibitors, as evidenced by many randomized clinical trials [39, 40], were the only type of medications that outperformed other candidates in score development, proving that their clinical application cannot be overemphasized. Higher NT-proBNP levels have been shown to be closely associated with pump failure death [41]. Our previous investigation also suggested that patients with higher NT-proBNP levels might derive less benefit from ICD [42]. In this study, log-transformed NT-proBNP showed a linear relationship with non-arrhythmic mortality; thus, quartiles of log-transformed values were given as cutoff points to appropriately quantify its hazard ratios.

## 5. Limitation

Given the retrospective design of this study, an overall evaluation of RV function was not possible. In addition, the lack of longitudinal echocardiographic data may have underestimated their prognostic role. However, the protocol used in the present study ensured that the echoes were comparable, and in our opinion, the timing just before implantation was a clinically relevant time for decision-making. It should be noted that this cohort comprising the Asian population may have limited generalizability to other ethnic groups; therefore, further validation should be performed. Angiotensin receptor neprilysin inhibitors and sodium glucose co-transporter 2 inhibitors are included in the current guidelines for optimal pharmacotherapy in patients with CHF. Studies in patients with up-to-date pharmacotherapy are needed to prove their efficacy in arrhythmic outcomes. The score developed in this study was determined using retrospectively collected data. Therefore, a prospective evaluation of this risk score as well as RV function is critical to confirm its accuracy and prognostic value.

## 6. Conclusions

Corresponding with previous studies, our observations supported the conceptual basis for the predictive value of RVOTD in a large cohort of CHF patients with heterogeneous heart diseases. Given the impact of RVOTD and other risk factors on VT/VF and non-arrhythmic mortality outcomes after implantable cardioverter-defibrillator therapy, a simple risk assessment tool incorporating RVOTD (RVOTD-ICD benefit score) could be generalized to both SCD primary and secondary prevention populations, thus optimizing the decision-making process for ICD implantation and interrogation.

## Data Availability

The datasets used and/or analyzed during the current study are available from the corresponding author on reasonable request.

## Supplementary Material

Supplementary material associated with this article can be found, in the online version, at https://doi.org/10.31083/j.rcm2409269.
