# Supplementary material for: Incremental Value of Right Ventricular Outflow Tract Diameter in Risk Assessment of Chronic Heart Failure Patients with Implantable Cardioverter Defibrillators: Development of RVOTD-ICD Benefit Score in Real-World Setting
Source: Rev Cardiovasc Med. 2023 Sep 22;24(9):269. doi: 10.31083/j.rcm2409269 (PMC11270099; doi:10.31083/j.rcm2409269)
Supplement: Supplementary file 1 [file 2153-8174-24-9-269-s1.zip › 2153-8174-24-9-269-s1.docx]

Supplementary material


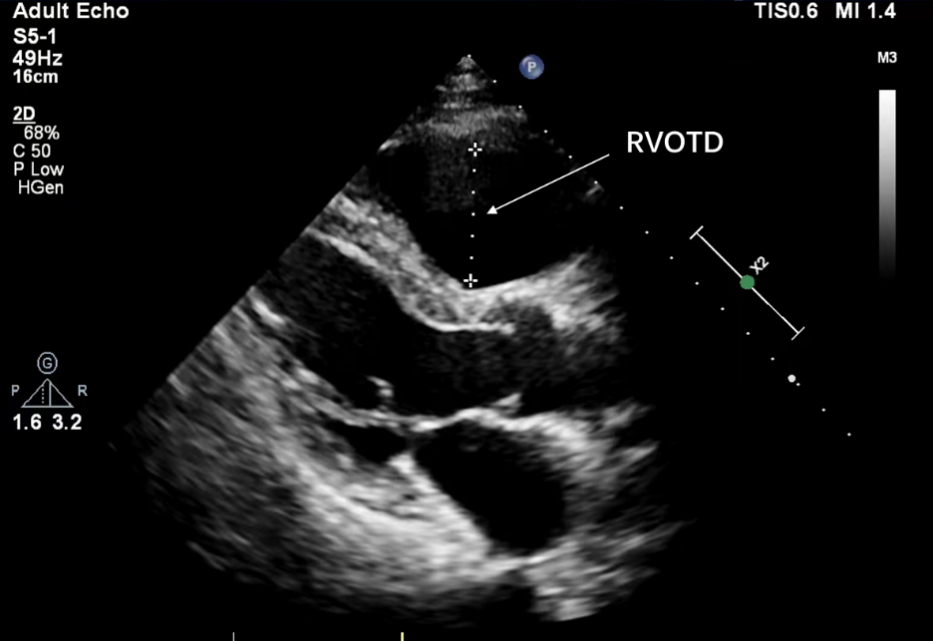


**Supplementary Fig. 1. Two-dimensional RVOTD measurements obtained from the parasternal long-axis (PSLAX) view in end-diastole [1].**


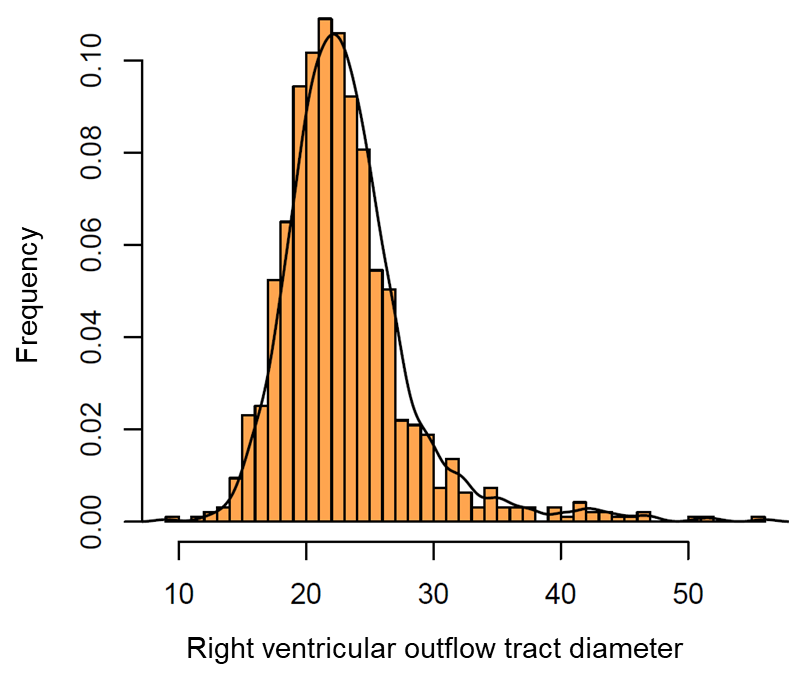


**Supplementary Fig. 2. Distribution of right ventricular diameter in the whole population.**

**Supplementary Fig. 3. Association between RVOTD and the risk of VT/VF in subgroups.**


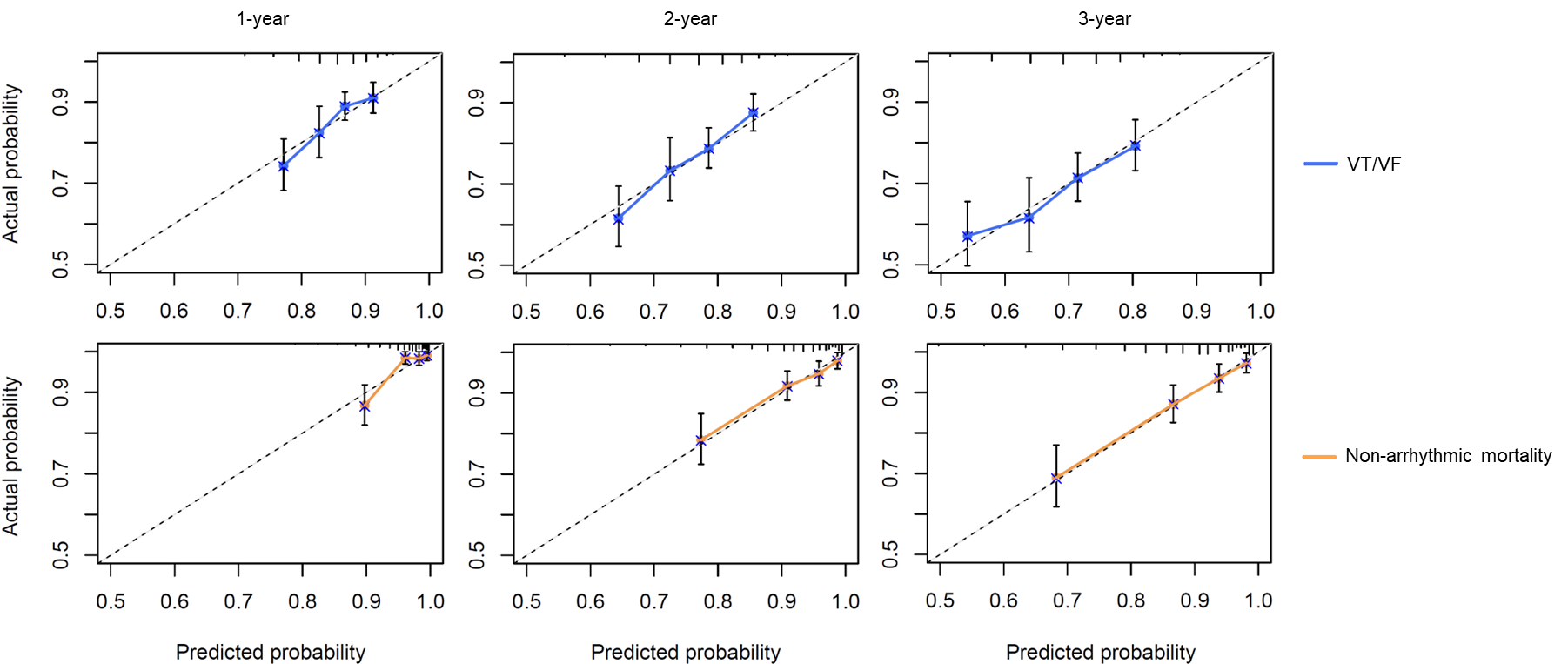


**Supplementary Fig. 4. Calibration curves for the predicted and actual probability of free from VT/VF and non-arrhythmic mortality by RVOTD-ICD risk score in 1, 2, and 3 years’ time.**


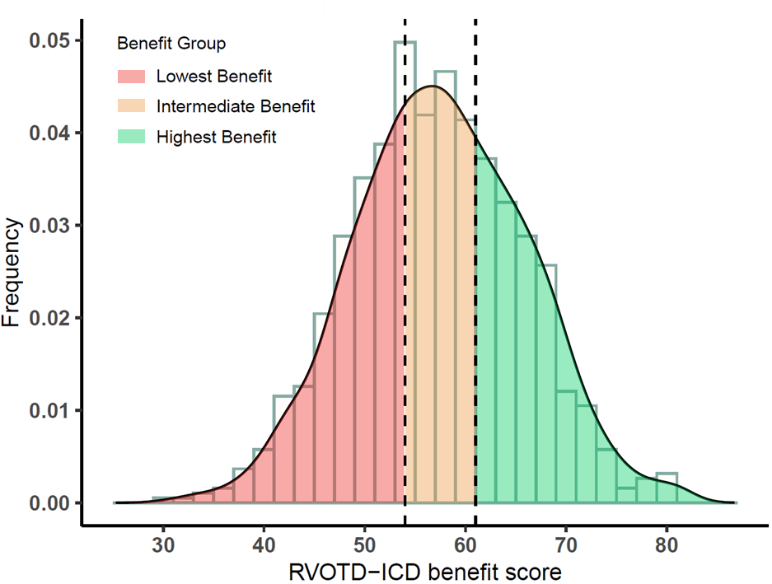


**Supplementary Fig. 5. Distribution of RVOTD-ICD benefit score in the whole population.**


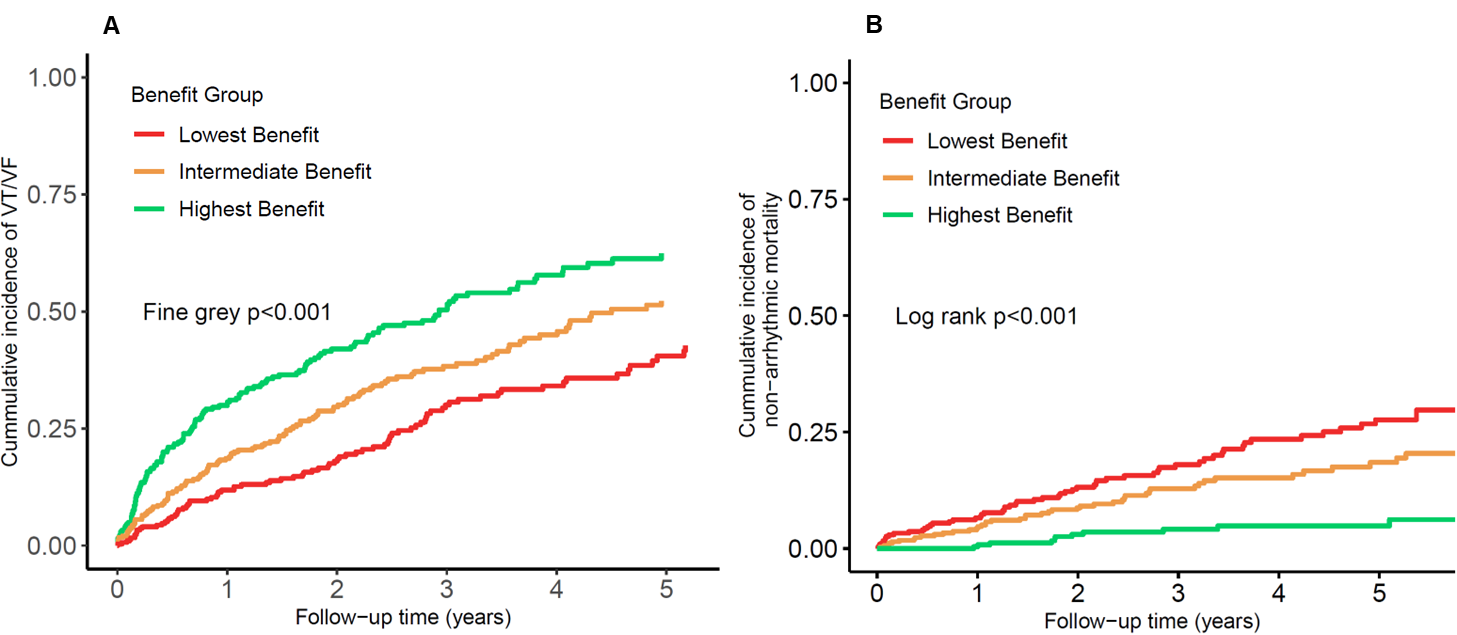


**Supplementary Fig. 6. Cumulative incidence of ventricular tachyarrhythmia (including anti-tachycardia pacing) (A) and Non-arrhythmic Mortality (B) among the three RVOTD-ICD benefit score groups.**


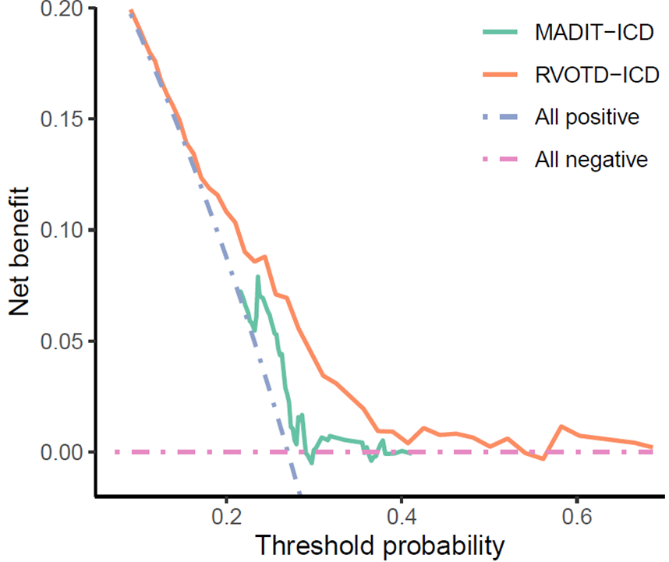


**Supplementary Fig. 7. Decision curve analysis for the MADIT-ICD and RVOTD-ICD benefit scores in 5 years’ all-cause mortality.**

Supplementary Table 1. Association of RVOTD and other possible risk factors with the risk of VT/VF and non-arrhythmic mortality.

| Parameters | VT/VF risk | | | | Non-arrhythmic Mortality | | | |
| --- | --- | --- | --- | --- | --- | --- | --- | --- |
|  | Univariable  analysis  HR (95% CI) | P value | Multivariable analysis  HR (95% CI) | P value | Univariable analysis  HR (95% CI) | P value | Multivariable  analysis  HR (95% CI) | P value |
| **Seattle proportional risk model** | | | | | | | | |
| Age (per decade older) | 0.87 (0.80-0.94) | <0.001 |  |  | 1.29 (1.12-1.48) | <0.001 |  |  |
| Male sex | 1.47 (1.08-2.01) | 0.016 |  |  | 0.88 (0.59-1.31) | 0.521 |  |  |
| Diabetes | 0.73 (0.53-1.02) | 0.068 |  |  | 1.73 (1.17-2.54) | 0.005 | 1.60 (1.09-2.37) | 0.017 |
| LV Ejection fraction (per 10% increase) | 0.95 (0.87-1.03) | 0.172 |  |  | 0.67 (0.58-0.77) | <0.001 |  |  |
| SBP (per 10 mmHg below 140) | 1.08 (1.00-1.08) | 0.049 |  |  | 1.15 (1.02-1.30) | 0.022 |  |  |
| Serum sodium level (each mEq/L below 145) | 1.02 (0.98-1.06) | 0.289 |  |  | 1.05 (0.99-1.11) | 0.102 |  |  |
| Creatinine (mg/dL) | 1.00 (0.99-1.01) | 0.952 |  |  | 1.00 (1.00-1.01) | 0.547 |  |  |
| NYHA I/II | 1.12 (0.88-1.43) | 0.358 |  |  | 0.29 (0.20-0.41) | <0.001 | 0.51 (0.34-0.77) | 0.001 |
| Body mass index (per 5 kg/m^2^ increase) | 1.00 (0.85-1.18) | 0.993 |  |  | 0.88 (0.69-1.12) | 0.308 |  |  |
| Digoxin use | 1.08 (0.83-1.40) | 0.579 |  |  | 1.80 (1.27-2.54) | <0.001 |  |  |
| **MADIT-ICD VT/VF score & MADIT-ICD non-arrhythmic mortality score** | | | | | | | | |
| Age<75 years | 2.27 (1.35-3.81) | 0.002 | 2.29 (1.36-3.86) | 0.002 | 0.51 (0.33-0.79) | 0.003 | 0.60 (0.38-0.95) | 0.030 |
| Male sex | As above |  |  |  | As above |  |  |  |
| SBP<140 mmHg | 1.29 (0.88-1.89) | 0.186 |  |  | 1.11 (0.66-1.88) | 0.685 |  |  |
| Heart rate>75 bpm | 1.23 (0.94-1.61) | 0.127 |  |  | 1.16 (0.78-1.72) | 0.457 |  |  |
| Arial arrhythmia | 1.09 (0.85-1.40) | 0.483 |  |  | 1.50 (1.06-2.12) | 0.023 |  |  |
| Myocardial infarction | 0.73 (0.57-0.94) | 0.013 |  |  | 1.14 (0.82-1.61) | 0.444 |  |  |
| prior NSVT | 1.62 (1.02-2.58) | 0.043 | 1.63 (1.02-2.61) | 0.039 |  |  |  |  |
| prior VT/VF | 1.77 (1.20-2.61) | 0.004 | 1.85 (1.25-2.72) | 0.002 | 0.58 (0.39-0.86) | 0.006 |  |  |
| LVEF≤25% | 1.09 (0.75-1.59) | 0.649 |  |  | 2.06 (1.32-3.20) | 0.001 |  |  |
| NYHA class≥II | 0.93 (0.67-1.29) | 0.660 |  |  | 2.43 (1.24-4.78) | 0.010 |  |  |
| Diabetes | As above |  |  |  | As above |  |  |  |
| Body mass index  <23 kg/m^2^ | 0.99 (0.77-1.27) | 0.901 |  |  | 1.28 (0.90-1.81) | 0.174 |  |  |
| **Other medications** | | | | | | | |  |
| RAAS inhibitors | 1.18 (0.91-1.53) | 0.215 |  |  | 0.65 (0.46-0.91) | 0.014 | 0.62 (0.44-0.88) | 0.008 |
| β-blocker | 0.84 (0.58-1.21) | 0.352 |  |  | 0.86 (0.51-1.45) | 0.577 |  |  |
| Diuretic | 1.34 (1.02-1.77) | 0.037 |  |  | 2.07 (1.30-3.29) | 0.002 |  |  |
| Mineralocorticoid  receptor antagonist | 1.33 (1.03-1.72) | 0.026 |  |  | 1.13 (0.78-1.62) | 0.517 |  |  |
| Digoxin | 1.08 (0.83-1.40) | 0.579 |  |  | 1.80 (1.28-2.53) | 0.001 |  |  |
| Antiarrhythmic drugs | 1.16 (0.91-1.48) | 0.222 |  |  | 0.98 (0.69-1.38) | 0.891 |  |  |
| **Echocardiographic parameters** | | | | | | | | |
| LVEDD | 1.14 (1.01-1.29) | 0.028 |  |  | 1.63 (1.38-1.93) | <0.001 | 1.39 (1.16-1.66) | <0.001 |
| LVPWT | 0.99 (0.93-1.06) | 0.858 |  |  | 0.98 (0.89-1.07) | 0.611 |  |  |
| IVST | 0.98 (0.95-1.01) | 0.267 |  |  | 0.91 (0.75-1.11) | 0.358 |  |  |
| LAD | 1.11 (0.99-1.24) | 0.080 |  |  | 1.47 (1.27-1.70) | <0.001 |  |  |
| RVOTD | 1.23 (1.12-1.35) | <0.001 | 1.22 (1.11-1.33) | 0.002 | 0.97 (0.82-1.13) | 0.680 |  |  |
| **Laboratory parameters** | | | | | | | | |
| ln (NT-proBNP) | 1.00 (0.80-1.23) | 0.952 |  |  | 5.16 (3.55-7.53) | <0.001 | 3.03 (2.00-4.58) | <0.001 |
| hs-cTnI (per 1 SD) | 0.92 (0.79-1.07) | 0.287 |  |  | 1.08 (1.00-1.17) | 0.053 |  |  |
| hs-CRP (per 1 SD) | 1.02 (0.91-1.14) | 0.763 |  |  | 1.29 (1.12-1.48) | <0.001 |  |  |

All continuous variables are included per SD unless specified otherwise. Single variable validated in more than one different risk models (e.g. age) only entered once into multivariable analysis. IVST, interventricular septal thickness; LVPWT, left ventricular posterior wall thickness; other abbreviations as in Table 1.

Supplementary Table 2. Association Between Right Ventricular Outflow Tract Diameter Levels and Outcomes (ATP included).

| Group | Event rate  (per 100 person-year) | Unadjusted  HR (95% CI) | p  value | SPRM adjusted^*^  HR (95% CI) | p  value | MADIT-ICD adjusted^†^  HR (95% CI) | p  value |
| --- | --- | --- | --- | --- | --- | --- | --- |
| VT/VF events | 16.89 (15.21-18.72) |  |  |  |  |  |  |
| Lower median | 13.83 (11.79-16.12) | Reference |  | Reference |  | Reference |  |
| Higher median | 20.60 (17.85-23.64) | 1.37 (1.11-1.68) | 0.003 | 1.33 (1.07-1.63) | 0.008 | 1.24 (1.00-1.53) | 0.046 |
| per 1 SD increase |  | 1.19 (1.09-1.29) | <0.001 | 1.14 (1.04-1.24) | 0.004 | 1.14 (1.04-1.24) | 0.005 |

*SPRM adjusted model initially included RVOTD and all the variables in Seattle Proportional Risk Model (male sex, younger age, no diabetes, lower left ventricular ejection fraction, systolic blood pressure, lower creatinine level, serum sodium level, better NYHA functional class, body mass index and digoxin use).

†For VT/VF events, MADIT-ICD adjusted model initially included RVOTD and all the variables in MADIT-ICD VT/VF score (LVEF<25%, atrial arrhythmia, heart rate >75 bpm, SBP <140 mmHg, myocardial infarction, age<75 years, male, and prior sustained VT/VF), which were then backward selected based on AIC rule; For non-arrhythmic mortality, MADIT-ICD adjusted model initially included RVOTD and all the variables in MADIT-ICD non-arrhythmic mortality score (NYHA≥II, diabetes, BMI<23kg/m^2^, atrial arrhythmia, LVEF≤25%, age≥75), which were then backward selected based on AIC rule.

Supplementary Table 3. Comparison of Area under Receiver Operating Characteristic Curve and Net Reclassification Improvement (NRI) for VT/VF and Non-arrhythmic Mortality between MADIT-ICD and RVOTD-ICD risk scores.

| Full cohort | VT/VF score | | | | Non-arrhythmic mortality score | | | |
| --- | --- | --- | --- | --- | --- | --- | --- | --- |
|  | AUC (95% CI) | P value | NRI (%, 95%CI) | P value | AUC (95% CI) | P value | NRI (%, 95%CI) | P value |
| 1-year |  |  |  |  |  |  |  |  |
| MADIT-ICD | 0.57 (0.52-0.62) | 0.029 | Ref. | 0.020 | 0.69 (0.60-0.77) | 0.006 | Ref. | <0.001 |
| RVOTD-ICD | 0.64 (0.59-0.70) |  | 16.1 (1.3-28.0) |  | 0.81 (0.73-0.88) |  | 42.4 (17.2-55.7) |  |
| 2-year |  |  |  |  |  |  |  |  |
| MADIT-ICD | 0.56 (0.51-0.60) | 0.008 | Ref. | 0.020 | 0.66 (0.60-0.72) | 0.007 | Ref. | <0.001 |
| RVOTD-ICD | 0.64 (0.59-0.68) |  | 16.3 (2.6-28.1) |  | 0.75 (0.69-0.81) |  | 31.7 (10.5-42.6) |  |
| 3-year |  |  |  |  |  |  |  |  |
| MADIT-ICD | 0.56 (0.51-0.61) | 0.103 | Ref. | 0.020 | 0.65 (0.59-0.71) | <0.001 | Ref. | <0.001 |
| RVOTD-ICD | 0.62 (0.57-0.66) |  | 12.1 (1.7-23.4) |  | 0.78 (0.73-0.83) |  | 39.5 (22.2-50.3) |  |

Supplementary Table 4. Observed VT/VF and non-arrhythmic mortality risk in primary and secondary prevention groups.

| RVOTD-benefit group | At 1 year |  | At 2 years |  | At 3 years |  |
| --- | --- | --- | --- | --- | --- | --- |
|  | VT/VF | Non-arrhythmic  Mortality | VT/VF | Non-arrhythmic  mortality | VT/VF | Non-arrhythmic  mortality |
| Primary prevention |  |  |  |  |  |  |
| observed rate (%) | 10.9 | 5.3 | 18.3 | 11.2 | 25.9 | 16.9 |
| 95% CI | 7.7-14.8 | 2.7-7.8 | 13.8-23.3 | 7.4-14.9 | 20.1-30.0 | 12.0-21.6 |
| Secondary prevention |  |  |  |  |  |  |
| observed rate (%) | 16.3 | 3.0 | 25.3 | 7.4 | 31.8 | 10.1 |
| 95% CI | 13.4-19.4 | 1.6-4.4 | 21.7-29.0 | 5.2-9.6 | 27.7-36.0 | 7.4-12.8 |

Supplementary Table 5. Net benefit of using MADIT-ICD and RVOTD-ICD benefit score for identifying all-cause mortality conditional on different decision thresholds.

| Decision threshold (%) | Net benefit vs all negative | | |
| --- | --- | --- | --- |
|  | All positive | MADIT-ICD | RVOTD-ICD |
| 20 | 0.073 | 0.072 | 0.103 |
| 25 | 0.025 | 0.059 | 0.071 |
| 30 | -0.042 | 0.001 | 0.045 |
| 35 | -0.132 | 0.004 | 0.019 |
| 40 | -0.217 | 0.001 | 0.004 |

Reference

1. Addetia K, Miyoshi T, Citro R, et al. Two-Dimensional Echocardiographic Right Ventricular Size and Systolic Function Measurements Stratified by Sex, Age, and Ethnicity: Results of the World Alliance of Societies of Echocardiography Study. Journal of the American Society of Echocardiography : official publication of the American Society of Echocardiography. 2021; 34: 1148-1157.e1141.
